# Summary of reference chemicals evaluated by the fish short‐term reproduction assay, OECD TG229, using Japanese Medaka, *Oryzias latipes*

**DOI:** 10.1002/jat.4104

**Published:** 2021-01-24

**Authors:** Yuta Onishi, Norihisa Tatarazako, Masaaki Koshio, Tetsuro Okamura, Haruna Watanabe, Atsushi Sawai, Jun Yamamoto, Hidenori Ishikawa, Tomomi Sato, Yukio Kawashima, Kunihiko Yamazaki, Taisen Iguchi

**Affiliations:** ^1^ Institute of Environmental Ecology IDEA Consultants, Inc. Yaizu Japan; ^2^ Department of Science and Technology for Biological Resources and Environment, Graduate School of Agriculture Ehime University Matsuyama Japan; ^3^ Center for Environmental Risk Research National Institute for Environmental Studies Tsukuba Japan; ^4^ Nanobioscience Yokohama City University Yokohama Japan; ^5^ Japan NUS Co. Tokyo Japan; ^6^ Environmental Health Department Ministry of the Environment Tokyo Japan

**Keywords:** endocrine disrupting effect, Japanese medaka, OECD TG229, *Oryzias latipes*, reproduction

## Abstract

Under the Organisation for Economic Co‐operation and Development (OECD), the Ministry of the Environment of Japan (MOE) added Japanese medaka (*Oryzias latipes*) to the test guideline fish short‐term reproduction assay (FSTRA) developed by the United States Environmental Protection Agency (US EPA) using fathead minnow (*Pimephales promelas*). The FSTRA was designed to detect endocrine disrupting effects of chemicals interacting with the hypothalamic–pituitary–gonadal axis (HPG axis) such as agonists or antagonists on the estrogen receptor (Esr) and/or the androgen receptor (AR) and steroidogenesis inhibitors. We conducted the FSTRA with Japanese medaka, in accordance with OECD test guideline number 229 (TG229), for 16 chemicals including four Esr agonists, two Esr antagonists, three AR agonists, two AR antagonists, two steroidogenesis inhibitors, two progesterone receptor agonists, and a negative substance, and evaluated the usability and the validity of the FSTRA (TG229) protocol. In addition, in vitro reporter gene assays (RGAs) using Esr1 and AR*β* of Japanese medaka were performed for the 16 chemicals, to support the interpretation of the in vivo effects observed in the FSTRA. In the present study, all the test chemicals, except an antiandrogenic chemical and a weak Esr agonist, significantly reduced the reproductive status of the test fish, that is, fecundity or fertility, at concentrations where no overt toxicity was observed. Moreover, vitellogenin (VTG) induction in males and formation of secondary sex characteristics (SSC), papillary processes on the anal fin, in females was sensitive endpoints to Esr and AR agonistic effects, respectively, and might be indicators of the effect concentrations in long‐term exposure. Overall, it is suggested that the in vivo FSTRA supported by in vitro RGA data can adequately detect effects on the test fish, *O. latipes*, and probably identify the mode of action (MOA) of the chemicals tested.

## Abstract 2

Fish short‐term reproduction assay (OECD, TG229) with Japanese medaka (*Oryzias latipes*) was conducted for 16 reference chemicals including 4 estrogen receptor (Esr) agonists, 2 Esr antagonists, 3 androgen receptor (AR) agonists, 2 AR antagonists, 2 steroidogenesis inhibitors, 2 progesterone receptor agonists, and a negative substance, and confirmed the usability and the validity of the TG229 protocol.

## 1 INTRODUCTION

The Ministry of the Environment of Japan (MOE) published its fourth program on endocrine disrupting effects of chemical substances (EDC) “EXTEND 2016” (MOE, 2016) of which the basic concepts and the framework were inherited from the preceding program “EXTEND 2010” (MOE, 2010), in June 2016. The EXTEND 2016 Program, which consists of Tier‐1 screening to evaluate endocrine disrupting potency and Tier‐2 testing to assess adverse endocrine disrupting effects on animals, has been progressing on the implementation of testing and assessment strategies, focusing on effects on reproduction (estrogen and androgen related), development (thyroid hormone related), and growth (juvenile and ecdysone hormones related). Regarding effects on reproduction, applying an in vitro reporter gene transcriptional assay (RGA) using estrogen receptor (Esr) and androgen receptor (AR) of Japanese medaka (*Oryzias latipes*), for example, the Esr1 (also known as ER*α*) and the AR*β*, and an in vivo fish short‐term reproduction assay (FSTRA) with Japanese medaka, are used in the Tier‐1 screening. Under the Organization for Economic Co‐operation and Development (OECD), the FSTRA and fish 21‐day assay, which the MOE had been involved in developing the protocols using Japanese medaka, were established as OECD TG229 (OECD, 2012a) and OECD TG230 (OECD, 2009a), respectively. TG229, for which the OECD had revised the test conditions for Japanese medaka based on the proposal from the MOE in 2012 (OECD, 2012a), recommends the use of three small fish species as test species, that is, fathead minnow (*Pimephales promelas*), zebrafish (*Danio rerio*), and Japanese medaka. In the EXTEND 2016 Program, the Medaka Extended One Generation Reproduction Test (MEOGRT), which has been adopted as OECD TG240, is conducted for candidate chemicals in which effects on fish reproduction were suspected based on the results of the Tier‐1 screening evaluation.

The FSTRA, conducted in accordance with the OECD TG229 includes the endpoints directly related to reproduction, that is, spawning status, and is designed to detect endocrine disrupting effects interacting with the hypothalamic–pituitary–gonadal axis (HPG axis), which may respond to substances that impact on it at different levels (OECD, 2012a). The literature on adverse effects on reproduction of fish caused by several estrogenic substances, such as 17*β*‐estradiol (E2), 17*α*‐ethynylestradiol (EE2), and alkylphenols, has seen numerous papers published since the late 1990s when EDC issues became apparent (Hara, Hiramatsu, & Fujita, 2016; Matthiessen, Wheeler, & Weltje, 2018; OECD, 2009b; Urushitani et al., 2007). Moreover, much research has been conducted to develop novel test methods, biomarkers and endpoints and select test species to assess various adverse effects that might be related to the endocrine system (Carnevali, Santangeli, Forner‐Piquer, & Basili, 2018; Manibusan & Touart, 2017; McArdle et al., 2020). On the other hand, there are studies in which multiple chemical substances in various modes of action (MOA) are comparably evaluated by a unified test species, conditions, and procedures, like a screening test in regulatory use. In the FSTRA, vitellogenin (VTG) protein, secondary sex characteristics (SSC), and reproductive status are quantitatively measured, and the Esr agonistic and antagonistic, AR agonistic and antagonistic, and steroidogenesis inhibitory potency of the test chemicals can be assessed based on the response in the endpoints (OECD, 2012a). The reproductive status, that is, fecundity and fertility during the exposure period, is the most important endpoint despite being measurable without high technical expertise. On the other hand, it was also suggested that fecundity can be influenced by nonchemical factors (presumably selection of smaller females, low food, and/or water temperature, but not specified in the report) in the validation report of the FSTRA using fathead minnow by the United States Environmental Protection Agency (US EPA, 2007). The FSTRA using fathead minnow is a key component of the US EPA's Endocrine Disruptor Screening Program (EDSP), which uses a weight‐of‐evidence analysis based on data from several assays to identify the potential for chemicals to act as agonists or antagonists of Esr or AR or inhibitors of steroidogenic enzymes (Ankley & Jensen, 2014), though it was pointed out that VTG and fecundity in the controls had high intralaboratory and interlaboratory variabilities, based on the data from 49 studies performed in the US EPA's EDSP, and the historical control data were of limited use during study interpretation (Wheeler, Valverde‐Garcia, & Crane, 2019). Because females of Japanese medaka produce 10–30 eggs daily in the conditions appropriately controlled (Ankley & Johnson, 2004; Flynn et al., 2017; Hirshfield, 1980; Koger, Teh, & Hinton, 1999; OECD, 2006b), the means of the daily fecundity during the FSTRA in controls are probably more stable compared with the other small test fish, for example, zebrafish and fathead minnow. Nevertheless, to practically and effectively use the FSTRA using Japanese medaka as Tier‐1 screening under the EXTEND 2016 Program, it is needed to evaluate intralaboratory and interlaboratory variabilities in control fish for endpoint measurements including reproductive status and to verify the applicability of the endpoints for screening of chemical effects by various MOAs.

This report describes the results from a study of the in vivo FSTRA using Japanese medaka in accordance with the OECD TG229 and in vitro reporter gene assay (RGA) using Esr and AR of Japanese medaka, for 16 substances. The 16 test chemicals, which were selected referring to the other validation studies and the literature describing EDCs in fish, included progesterone receptor (PR) agonists known from mammals and a substance in which negative effects on the endocrine system have been suspected (negative substance), as well as Esr agonists and antagonists, AR agonists and antagonists, and steroidogenesis inhibitors. These reference chemical studies were conducted with the financial support of the MOE, in the process of establishing the test guideline for Japanese medaka and to obtain the knowledge and the data to support the Tier‐1 evaluation under the EXTEND 2016 Program.

## 2 MATERIALS AND METHODS

### 2.1 Test chemicals

#### 2.1.1 Esr agonists

As Esr agonists, four chemicals were used. E2 is an endogenous estrogen in vertebrates and has been frequently used in several validation studies of fish testing, for example, the Medaka multigeneration test (MMT), which is the original method of MEOGRT (Flynn et al., 2017; OECD, 2015; US EPA, 2013), the 21‐Day Fish Screening Assay (OECD, 2006a, 2006b), and the Fish Sexual Development Test (FSDT) (OECD, 2011c). EE2 is a synthetic estrogen that, like E2, has been the most widely used in fish studies, as a reference chemical of a strong Esr agonist (OECD, 2009b). An alkylphenol, 4‐*tert*‐pentylphenol (PTH) has weak estrogenic activity in fish and was used for the OECD validations for the 21‐day Fish Assay and FSDT (OECD, 2006b, 2011a). 4‐Chloro‐3‐methylphenol (CMP) was used in the validation of the MMT, as a weak estrogenic substance (Flynn et al., 2017; US EPA, 2015). A study suggested that CMP bound to both the recombinant human and rainbow trout (*Oncorhynchus mykiss*) Esrs and induced rainbow trout VTG mRNA at about the same concentrations as overt toxicity (Schmieder et al., 2004; US EPA, 2013).

#### 2.1.2 Esr antagonists

Two chemicals, tamoxifen citrate (TAM) and raloxifene hydrochloride (RAL), were used for the Esr antagonist studies. Both chemicals are selective Esr modulators, which have features that can act as Esr agonists and antagonists depending on the target tissues in mammals (An, 2016; Shang & Brown, 2002), and are used for treatment of breast cancer, osteoporosis, and postmenopausal symptoms. Regarding TAM effects on fish, a decrease in VTG and fecundity was reported from the partial life cycle assay with female zebrafish (van der Ven, van den Brandhof, Vos, & Wester, 2007).

#### 2.1.3 AR agonists

As AR agonists, three chemicals were used. 17*β*‐trenbolone (TRB) and 17*α*‐methyltestosterone (MT) are synthetic anabolic‐androgenic steroids. For example, TRB is used as a subcutaneous implant for growth promotion in beef cattle (Ankley et al., 2003). In the validation for the 21‐day Fish Assay, FSTRA with fathead minnow and MMT (MEOGRT), TRB was used as an AR agonist test chemical (Flynn et al., 2017; OECD, 2009a; US EPA, 2007, 2013). MT is also commonly used to validate fish assays (OECD, 2009b). Though 11‐ketotestosterone (11KT) is an endogenous androgen in fish species, because of the cost of this androgen, 5*α*‐dihydrotestosterone (DHT), produced from testosterone by 5*α*‐reductase in mammals, was used for the validation of the FSDT (OECD, 2012b).

#### 2.1.4 AR antagonists

Two chemicals, flutamide (FLT) and vinclozolin (VCZ), were used as AR antagonists. FLT is an antiandrogenic medication for prostate cancer and was used in the validation of the 21‐day Fish Assay and FSDT (OECD, 2006a, 2012b). VCZ, a dicarboximide fungicide, was used in studies of effects on reproduction in medaka (Kiparissis, Metcalfe, Balch, & Metcalfe, 2003), guppy (*Poecilia reticulata*) (Bayley, Larsen, Baekgaard, & Baatrup, 2003), and fathead minnow (Makynen et al., 2000), and the validation of the FSTRA with fathead minnow (US EPA, 2007) and MEOGRT (Flynn et al., 2017; US EPA, 2013). As for VCZ, some literature reported that its metabolites, M1 and M2, had an antiandrogenic effect on male rat and fish as well as VCZ itself (Baatrup & Junge, 2001; Gray, Ostby, & Kelce, 1994; Makynen et al., 2000). Both the chemicals were also used in the validation studies for the screening assays that specialized in detecting antiandrogenic effects using female three‐spined stickleback (*Gasterosteus aculeatus*) or juvenile medaka (Nakamura et al., 2014; OECD, 2010).

#### 2.1.5 Steroidogenesis inhibitors

Ketoconazole (KCZ) and prochloraz (PCL), which are known as azole‐based fungicides that inhibit the synthesis of ergosterol, a vital component of the fungal cell membrane (Zarn, Bruschweiler, & Schlatter, 2003), were used as steroidogenesis inhibitors. Several literatures have reported that these chemicals inhibited directly and/or consequently the activity of several cytochrome P450 enzymes, including aromatase, involved in steroidogenesis in vivo and/or in vitro (Andersen, Vinggaard, Rasmussen, Gjermandsen, & Bonefeld‐Jorgensen, 2002; Blystone et al., 2007; Monod, De Mones, & Fostier, 1993; Skolness et al., 2011; Villeneuve et al., 2007). Both the chemicals were used in the validation study of the FSTRA using fathead minnow by the US.EPA (Ankley et al., 2005; US EPA, 2007), and PCL was also used to validate the FSDT and the MEOGRT (Flynn et al., 2017; OECD, 2011b).

#### 2.1.6 PR agonists

A natural progesterone (P4) and a synthetic progestin, levonorgestrel (LNG), were subjected to the assay to assess the applicability of the FSTRA protocol to the effects of PR agonists on the test species (medaka). P4 is an endogenous steroid involved in the menstrual cycle, pregnancy, and embryogenesis in mammals and LNG is a second‐generation progestin derived from 19‐nortestosterone (Lorenz et al., 2011). Because these two chemicals have been used as a component of oral contraceptives, they have been detected in the environment, in water (Kumar et al., 2015; Orlando & Ellestad, 2014; Shen, Chang, Sun, Wang, & Wu, 2018). Zeilinger et al. (2009) and Runnalls, Beresford, Losty, Scott, and Sumpter (2013) reported that synthetic progestin had affected reproduction of wild fish at environmental levels.

#### 2.1.7 Negative (inactive) substance

To validate the protocols of fish short‐term assays, a few substances, such as *n*‐octanol, potassium permanganate, and sodium dodecyl sulfate (SDS), had been previously used, but problematic results, such as considerable decreases in measured concentration and unexpectedly high fish mortality, were observed in these experimental studies (OECD, 2007, 2010; US EPA, 2007). In the present study, SDS was selected as a negative substance because the dilution water had a relatively low degree of hardness, for example, in 50 mg/L as CaCO_3_, which was expected to contribute to the stability of test concentration and the suppression of mortality by excessive toxicity.

For all the test chemicals, commercially available reagents with the highest degree of purity were obtained. The details of the reagents tested in the in vivo FSTRA and the in vitro RGA studies are shown in Table 1.

**TABLE 1:** Test chemicals and supplier and purity of chemical reagents used in FSTRA and RGA

Test chemical		CAS no.	Supplier	Purity (%)
Esr agonist	17*β*‐Estradiol (E2)	50‐28‐2	WAKO^a^, FUJI^b^	100^a^, 97.1^b^
	17*α*‐Ethynylestradiol (EE2)	57‐63‐6	TCI	99.8^a^, 99.7^b^
	4‐Chloro‐3‐methylphenol (CMP)	59‐50‐7	TCI	99.9^a^, 99.9^b^
	4‐*tert*‐Pentylphenol (PTH)	80‐46‐6	TCI	>98^a^, 99.7^b^
Esr antagonist	Tmoxifen citrate (TAM)	54965‐24‐1	WAKO	>98^a^, 99.8^b^
	Raloxifene hydrochloride (RAL)	82640‐04‐8	WAKO	>99^a^, 99.5^b^
AR agonist	5*α*‐Methyltestosterone (MT)	58‐18‐4	WAKO^a^, TCI^b^	99.6^a^, 99.8^b^
	5*α*‐Dihydrotestosterone (DHT)	521‐18‐6	TCI	99.0^a^, 99.7^b^
	17*β*‐Trenbolone (TRB)	10161‐33‐8	WAKO	>98^a^, 99.7^b^
AR antagonist	Flutamide (FLT)	13311‐84‐7	SIGMA^a^, WAKO^b^	>99^a^, 99.3^b^
	Vinclozolin (VCZ)	50471‐44‐8	SIGMA^a^, WAKO^b^	99.5^a^, 99.9^b^
Steroidogenesis inhibitor	Ketoconazole (KCZ)	65277‐42‐1	TCI	98.0^a^, 99.5^b^
	Prochloraz (PCL)	67747‐09‐5	SIGMA^a^, WAKO^b^	98.6^a^, 99.2^b^
PR agonist	Progesterone (P4)	57‐83‐0	WAKO^a^, TCI^b^	>98^a^, 99.8^b^
	Levonorgestrel (LNG)	17489‐40‐6	TCI	98.9^a^, 97.7^b^
Negative	Sodium dodecyl sulfate (SDS)	151‐21‐3	TCI	99.9^a^, 100^b^

Abbreviations: AR, androgen receptor; Esr, estrogen receptor; FUJI, FUJIFILM Wako Pure Chemical Corporation; FSTRA, fish short‐term reproduction assay; RGA, reporter gene assay; SIGMA, Sigma‐Aldrich Co. LLC; TCI, Tokyo Chemical Industry Co., Ltd.; WAKO, Wako Pure Chemical Industries, Ltd.

^a^ Reagents used in fish short‐term reproduction assay.

^b^ Reagents used in reporter gene assay.

### 2.2 Fish short‐term reproduction assay

The experimental studies on the FSTRA for the 16 chemicals were conducted in two laboratories in Japan, the National Institute for Environmental Studies (NIES, Lab‐1) and the Institute of Environmental Ecology, IDEA Consultants Inc. (Lab‐2), in accordance with OECD TG229 (OECD, 2012a) (Table 1).

#### 2.2.1 Test fish

The NIES‐R strain of Japanese medaka (*O. latipes*), one of the orange‐red varieties, was used in all the 16 chemical studies. For the studies, healthy and mature male and female medakas at 16 ± 2 weeks old were selected from a single‐stock population, which were bred within the test facilities. The test fish were maintained in conditions similar to the assay for at least 7 days, and spawning status was checked during the acclimation period. On the day the chemical exposure commenced, to ensure balanced distribution of test fish between the treatment groups for the reproductive status of the fish, the replicate vessels (fish tanks), with each containing three male and female medaka, were distributed to each treatment and control group, for example, by a randomized block design based on the number of fertilized eggs laid in the last 5–7 days during the acclimation, in each assay.

#### 2.2.2 Test concentrations

For each test chemical, three or four concentrations were set by reference to the results of previous validation studies and toxicity tests with Japanese medaka (Table 2). To prevent excessive lethal effects, the highest test concentration was determined based on acute toxicity (e.g., one third or tenth of the 96 h LC_50_). The water solubility of the test chemical was also taken into consideration when deciding the highest test concentration. To set the lower concentrations, a spacing factor ranging between two and five was used. All assays included a dilution water control (DWC).

**TABLE 2:** Nominal and measured test concentrations for test chemicals in FSTRA

Test chemicals		Test concentrations^a^			
		Conc. 1	Conc. 2	Conc. 3	Conc. 4
E2	Nominal (ng/L)	22	110	550	na
	Measured (ng/L)	22.1 (100%)	115 (105%)	553 (101%)	
EE2	Nominal (ng/L)	17	85	425	na
	Measured (ng/L)	17.8 (105%)	84.9 (100%)	424 (100%)	
CMP	Nominal (μg/L)	120	380	1,200	na
	Measured (μg/L)	108 (90%)	350 (92%)	1,060 (88%)	
PTH	Nominal (μg/L)	100	320	1,000	na
	Measured (μg/L)	96.5 (97%)	342 (92%)	1,100 (110%)	
TAM	Nominal (μg/L)	10	32	100	na
	Measured (μg/L)	10.0 (100%)	28.0 (88%)	83.3 (83%)	
RAL	Nominal (μg/L)	64	200	640	2,000
	Measured (μg/L)	63.9 (100%)	203 (100%)	721 (113%)	2,080 (104%)
MT	Nominal (ng/L)	20	80	320	na
	Measured (ng/L)	20.1 (101%)	77.4 (97%)	300 (94%)	
DHT	Nominal (μg/L)	0.1	0.32	1.0	3.2
	Measured (μg/L)	0.063 (63%)	0.26 (82%)	1.03 (103%)	2.97 (93%)
TRB	Nominal (ng/L)	10	32	100	320
	Measured (ng/L)	8.03 (80%)	26.8 (84%)	84.6 (85%)	291 (91%)
FLT	Nominal (μg/L)	125	250	500	1,000
	Measured (μg/L)	119 (95%)	248 (99%)	497 (99%)	925 (93%)
VCZ	Nominal (μg/L)	20	64	200	640
	Measured (μg/L)	14.5 (73%)	43.9 (69%)	137 (69%)	453 (71%)
KCZ	Nominal (μg/L)	125	250	500	1,000
	Measured (μg/L)	105 (84%)	233 (93%)	405 (81%)	795 (80%)
PCL	Nominal (μg/L)	12.5	25	50	na
	Measured (μg/L)	10.3 (82%)	20.6 (82%)	44.9 (90%)	
P4	Nominal (μg/L)	15.6	50	156	500
	Measured (μg/L)	11 (71%)	24 (48%)	64 (41%)	243 (49%)
LNG	Nominal (ng/L)	8.0	40	200	na
	Measured (ng/L)	7.3 (91%)	42.2 (106%)	226 (113%)	
SDS	Nominal (mg/L)	1.0	3.2	10	na
	Measured (mg/L)	0.748 (75%)	2.46 (77%)	10.0 (100%)	

Abbreviations: CMP, 4‐chloro‐3‐methylphenol; DHT, 5*α*‐dihydrotestosterone; E2, 17*β*‐estradiol; EE2, 17*α*‐ethynylestradiol; FLT, flutamide; FSTRA, fish short‐term reproduction assay; KCZ, ketoconazole; LNG, levonorgestrel; MT, 5*α*‐methyltestosterone; na, not available (because the assay was carried out in three concentrations); P4, progesterone; PCL, prochloraz; PTH, 4‐*tert*‐pentylphenol; RAL, raloxifene hydrochloride; SDS, sodium dodecyl sulfate; TAM, tamoxifen citrate; TRB, 17*β*‐trenbolone; VCZ, vinclozolin.

^a^ The percentages of the mean measured concentrations (*n* = 4) to the nominal are given in parentheses.

Test solutions at the selected concentrations were prepared by diluting a stock solution, which was prepared by an adequate method depending on the physicochemical properties of the test chemicals, for example, by simply mixing the test chemical in dilution water by stirring and/or sonicating for an appropriate time or by using a solid–liquid saturator. In the two laboratories, flow‐through exposure systems, in which a series of test solutions at the target concentrations can be continuously prepared and delivered to test vessels at controlled flow rates (Haselman et al., 2016; Watanabe et al., 2017), were used for the exposure experiments. Dechlorinated tap water in which the water quality had been checked at appropriate frequency was used as dilution water to prepare the test solutions and as a test solution for the controls.

#### 2.2.3 Chemical analysis

The chemical concentrations of test solutions, including DWC, were quantitatively measured once a week during the exposure period. The water samples, collected from the test vessels, were immediately subjected to chemical analysis or stored at 4°C until analysis. If there was a need to derive the limit of quantification (LOQ) required, a suitable pretreatment procedure, for example, solid–liquid extraction, was applied to the water samples. The analytical method and LOQ in each study are summarized in Table 3.

**TABLE 3:** Methods and LOQs for test solution analysis in FSTRAs

Test chemical	Sample pretreatment	Analysis	LOQ
E2	Solid–liquid extraction	LC–MS/MS	0.1 ng/L
EE2	Solid–liquid extraction	LC–MS/MS	0.4 ng/L
CMP	Solid–liquid extraction plus derivatization	GC–MS	3 μg/L
PTH	Liquid–liquid extraction	GC–MS	0.5 μg/L
TAM	Solid–liquid extraction	LC–MS	0.5 μg/L
RAL	None	LC–MS	5 μg/L
MT	Solid–liquid extraction	LC–MS/MS	0.5 ng/L
DHT	Solid–liquid extraction	LC–MS/MS	5 ng/L
TRB	Solid–liquid extraction	LC–MS	0.2 ng/L
FLT	None	LC–MS	0.01 μg/L
VCZ	Solid–liquid extraction	GC–MS	3 μg/L
KCZ	None	LC–MS/MS	1 μg/L
PCL	None	LC–MS	0.1 μg/L
P4	None	LC–MS	0.1 μg/L
LNG	Solid–liquid extraction	LC–MS/MS	0.9 ng/L
SDS	None	LC–MS	0.04 mg/L

Abbreviations: CMP, 4‐chloro‐3‐methylphenol; DHT, 5*α*‐dihydrotestosterone; E2, 17*β*‐estradiol; EE2, 17*α*‐ethynylestradiol; FLT, flutamide; FSTRA, fish short‐term reproduction assay; GC–MS, gas chromatography–mass spectrometer; KCZ, ketoconazole; LC–MS, liquid chromatograph–mass spectrometer; LC–MS/MS, liquid chromatograph–tandem mass spectrometer, LNG, levonorgestrel; LOQ, limit of quantification; MT, 5*α*‐methyltestosterone; P4, progesterone; PCL, prochloraz; PTH, 4‐*tert*‐pentylphenol; RAL, raloxifene hydrochloride; SDS, sodium dodecyl sulfate; TAM, tamoxifen citrate; TRB, 17*β*‐trenbolone; VCZ, vinclozolin.

#### 2.2.4 Chemical exposure and observation

In the FSTRA, three mature adult males and females in a test vessel were exposed together to the test chemicals in flow‐through condition for 3 weeks. In all the assays, each treatment group, including the DWC group, contained four replicate tanks. During the exposure period, the fish were fed a sufficient amount of live brine shrimp nauplii (newly hatched *Artemia* sp.) for daily spawning *ad libitum*. Fecal material in the tanks was appropriately removed by siphoning after feeding. Water temperature of test solutions was recorded for at least one vessel in each treatment and control group every day and for all vessels at least once a week. Furthermore, dissolved oxygen and pH of test solutions were measured for all test vessels at least once a week. Mortality and abnormal behavior and appearance in the fish exposed to test chemicals were daily observed, and any fish that died were removed from the tank as soon as possible. To assess the reproductive status, all eggs females spawned were collected every day. At the completion of the chemical exposure, surviving fish in each tank were sampled.

All of the chemical exposures were conducted in the conditions shown in Table 4 and satisfied the test acceptance criteria provided by the OECD TG229, except for the SDS study. In the SDS study, the dissolved oxygen level of test solution at the highest concentration dropped below 60% of saturation for a few days, due to microbial growth. However, it is considered that this problem did not have a significant impact on the test fish, because no mortality or any abnormal appearance or behavior was observed during this period.

**TABLE 4:** Test conditions for fish short‐term reproduction assay (FSTRA)

Exposure type	Flow through
Exposure duration	21 days
Test vessel	All‐glassware tank (approx. 7.8 [Lab 1] or 3 L [Lab 2] capacity)
Test solution volume in a tank	5 (Lab 1) or 2 L (Lab 2)
Number of test fish	6 fish (3 males and 3 females) per tank
Flow rate of test solution	20 ml/min (Lab 1) or 14 mL/min (Lab 2) (approx. 6 [Lab 1] or 10 [Lab 2] volume renewal/day)
Temperature of test solution	25 ± 1°C^a^
Dissolve oxgen in test solution	More than 60% of saturation^b^
Photoperiod	16‐h light and 8‐h dark
Feeding	Live brine shrimp (<24‐h old nauplii)
	*Ad libitum* ^c^
	Twice (Lab 1) or 3 times (Lab 2) a day (weekday) or once (Lab 1) or twice (Lab 2) a day (weekend)

^a^ The OECD test guideline prescribes 25 ± 2°C, but the measurements of temperature of test solutions ranged within 25 ± 1°C in all the 16 studies.

^b^ The saturation of dissolved oxygen dropped to below 60% for 1 or 2 days in the tanks at the highest concentration in SDS study.

^c^ All tanks were given the same amount of brine shrimp slurry in each day.

#### 2.2.5 Endpoint measurements

##### Spawning status (fecundity and fertility)

The eggs females released on the bottom of the tank were collected by siphoning, and the egg clutches still on female abdomen were carefully picked from the body of the females captured using a small net. All the eggs collected were microscopically observed and separately counted for fertilized and unfertilized. As an endpoint regarding fecundity, the mean of the number of the total of fertilized and unfertilized eggs (/female/day) was determined, and the fertility rate, which was defined as the ratio (percentage) of the number of fertilized eggs to the number of total eggs over the 21‐day exposure period, was calculated.

##### Necropsy and sample collection

The fish surviving at the completion of the exposure were anesthetized in ice‐cold water and then were dissected and sampled whole liver after measuring body length and weight. The liver samples were weighed and immediately stored under −20°C or less, until VTG quantification. In addition, the anal fin was imaged in a flat and spread‐out condition for each fish, or the posterior region of the fish including the anal fin was collected and stored in appropriate fixative, for example, 10% neutral buffered formalin, prior to SSC assessment.

##### Hepatic VTG

The concentrations of VTG in liver samples were quantitatively determined by the enzyme‐linked immunoadsorbent assay (ELISA) methods (OECD, 2012a; Tatarazako, Koshio, Hori, Morita, & Iguchi, 2004). Briefly, the liver samples were homogenized with the assay buffer included in the ELISA kit used. The homogenates in microtubes were centrifuged, and the supernatant (hepatic extract) was collected. The VTG concentrations in the hepatic extractions were quantitatively determined using commercially available ELISA kits, EnBio Medaka VTG ELISA (EnBio Tec Laboratories Co. Ltd., Tokyo, Japan) or medaka VTG ELISA assay kit (Trans Genic Inc., Fukuoka, Japan). In each of the 16 studies, the VTG ELISA kits within the same lot were used to eliminate an interlot variation. The limit of determination was 1.0 ng/mg of liver weight in all the VTG analyses.

##### Secondary sex characteristics

For SSC, the number of joint plates in which papillary processes (PPs) were visibly formed was counted on the images of anal fins or on the fixed samples under a microscope (Nakamura et al., 2014; OECD, 2012a).

#### 2.2.6 Statistical analysis for FSTRA data

For each endpoint, differences between the chemical treatment and the DWC were statistically analyzed in the replicate means basis (OECD, 2006c). Briefly, first the homogeneity of variance was assessed by Leven's or Bartlett's test, and then the data in which homogeneity of variance was confirmed were subjected to one‐way analysis of variance (ANOVA) followed by Dunnett's test. The data in which the assumption of homogeneity of variance was rejected were appropriately transformed (e.g., by log transformation, square root transformation, or arcsine transformation) and reanalyzed for homogeneity of variance. If no homogeneity of variances was found, even in the transformed data, the data were analyzed by nonparametric Kruskal–Wallis test followed by Steel's test or Dunn's test. A *p* value less than 0.05 was considered significant for all the statistical analyses.

Based on the results from the statistical analysis, the lowest observed effect concentration (LOEC) was determined in each endpoint. The LOEC was defined as the lowest tested concentration in which a significant effect (decrease or increase) compared with the control was observed and equal to or greater effects were found in all the concentrations higher than that (OECD, 2015).

### 2.3 In vitro RGA

To support interpretation of the biological effects observed in the FSTRA studies, in vitro RGAs using medaka Esr1 (mEsr1) and medaka AR*β* (mAR*β*) were performed for the 16 test chemicals. In the present study, Esr1 and AR*β*, which are ancestral subtypes among Esrs and ARs in Japanese medaka, were selected based on the phylogenetical analyses of Esr and AR with each receptor RGAs (Ogino et al., 2016, 2018; Tohyama et al., 2015).

#### 2.3.1 Medaka Esr1 RGA

The mEsr1 RGA consisting of agonist and antagonist assays were performed according to the methods previously reported (Katsu et al., 2010; Lange et al., 2012; Miyagawa et al., 2014). Briefly, human embryonic kidney cells 293 (HEK293), precultured, were seeded in 96‐well plates at 15,000 cells/well with 200 μl of culture medium, phenol‐red‐free Dulbecco's Modified Eagle's medium (ThermoFisher Scientific K.K., Tokyo, Japan) supplemented with 2‐mM l‐glutamine and 10% charcoal/dextran‐treated fetal bovine serum (Hyclone, UT, USA), and then incubated for 24 h in 5% CO_2_ at 37°C. After the 24‐h incubation, 20 μl of test medium containing 40 ng of medaka Esr1/pcDNA3.1, 80 ng of ERE‐TK‐Luc, and 20 ng of pRL‐TK‐Rlu and 1.2 μL of transfection reagent FuGENE HD (Promega, WI, USA) were added into each well. The assay plates were incubated for another 4 h in order to transfect the three vectors into the HEK293 cells. After the vectors were successfully transfected, in agonist assay, 24 μl of concentrated test chemical solution was applied to the test medium in the plate wells. In antagonist assay, the natural Esrt ligand E2 (FUJIFILM Wako Pure Chemical Corporation, Osaka, Japan, purity 97.1%) was spiked into the test medium at 1 nM together with test chemicals. After chemical treatment for 40 h, luciferase activity of the cells was measured, and the promoter activity was calculated as firefly (*Photinus pyralis*)‐/sea pansy (*Renilla reniformis*)‐luciferase activity in each well. The luciferase activities were quantified by a chemiluminescence assay with Dual‐Luciferase Reporter Assay System (Promega) in accordance with the manufacturer's instructions, and luminescence intensity of firefly and sea pansy luciferin was measured using a luminometer, TriStar LB941 (Berthold Technology, Oak Ridge, TN, USA). In the agonist and the antagonist assays, E2 and 4‐hydroxytamoxifen (4HTAM; Sigma‐Aldrich, MO USA, purity 99.9%) were used as positive controls, respectively, to confirm the assays adequately worked.

#### 2.3.2 Medaka AR*β* RGA

The mAR*β* RGA in which the agonist and the antagonist assays were performed as same as the mEsr1 RGA was carried out according to the methods previously reported by Lange et al. (2015). For the assays, human liver cancer cell line, HepG2, was used as the host cells, and mAR*β*/pcDNA3.1, MMTV‐Luc, and pRL‐TK‐Rlu were used as mAR*β* expression vector, reporter vector, and internal control vector, respectively. Other materials and methods, for example, operating procedure, reagents, and incubation conditions, were almost the same as the mEsr1 RGA previously described beside that 11KT (Sigma‐Aldrich, purity 99.0%), a main AR ligand in fish, was competitively spiked at 50 nM into the test medium with test chemicals in antagonist assay. In the agonist and the antagonist assays, 11KT and 2‐hydroxyflutamide (2HFLT; Sigma‐Aldrich, purity 98.5%) were used as positive controls, respectively.

#### 2.3.3 Estimation of effect concentration

Effect concentrations, that is, EC_50_ (a half maximal effective concentration) in the agonist assay and IC_50_ (a half maximal inhibitory concentration) in the antagonist assay, were estimated by nonlinear curve fitting (e.g., three parameters logistic regression curve) using GraphPad Prism (GraphPad Software Inc., La Jolla, CA, USA).

## 3 RESULTS

### 3.1 Fish short‐term reproduction assay

#### 3.1.1 Measured concentrations of test chemicals

The mean measured concentrations of test chemicals in test solutions ranged within ±20% of the nominal concentrations in all the studies, except DHT, VCZ, P4, and SDS studies (Table 2). The measured concentrations were slightly below 80% of the nominal at the lowest concentration for DHT, in all four test concentrations of the VCZ study, and at the lowest and middle concentrations in the SDS study.

In the P4 study, a remarkable decrease of P4 concentration was found in the chemical analysis conducted on the 13th day of the exposure (in the second week) for all the exposure concentrations. Because the cause was considered to be biodegradation due to the microbial growth, cleaning of the inside of the exposure system, for example, dilution tanks, solution supply pipes, and exposure aquariums, was conducted, and at the same time, the cause was verified. When the test solutions prepared in the system were sampled in another glass tanks and treated with ultraviolet (UV) irradiation, the P4 concentration after 24‐h UV treatment maintained more than 80% of the nominal, whereas the P4 concentration in the sample without UV treatment was decreased to less than 20% of the nominal suggesting biodegradation (data not shown). Though the extreme reduction of P4 concentrations were solved within 2 or 3 days by the device cleaning, as a result, the time‐weighted means of measured P4 concentrations less than 50% of the nominal were suggested for all, except the lowest, the exposure concentrations.

#### 3.1.2 Mortality

The results of the FSTRA studies, that is, the mean of endpoint measurements for each treatment and control fish, are summarized in Table 5. The other measurement data but not included in apical endpoints in OECD TG229, for example, lengths, weights, hepatosomatic indices (HSI), and gonadosomatic indices (GSI) of the exposed fish, are provided in Table 6.

**TABLE 5:** Mortality, hepatic VTG concentration, SSC, number of total and fertilized eggs, and fertility rate in FSTRAs

Test chemical		Measured	Mortality (%)		VTG (ng/mg of liver)				SSC					Number of			Number of fertilized				Fertility rate	
		conc.	Male	Female		Male		Female	Male		Female			total eggs			eggs				(%)	
Esr agonist	E2	Control	0	0		0.5 ± 0.0		2,460 ± 241		107 ± 13		0 ± 0		23 ± 0.9		22	±	1.1		97	±	1.2
		22.1 ng/L	0	0	↑	424 ± 379		3,420 ± 991		109 ± 10		0 ± 0		20 ± 2.9	↓	18	±	1.6		90	±	5.3
		115 ng/L	8	8	↑	13,400 ± 1900	↑	5,500 ± 1,370		112 ± 10		0 ± 0		22 ± 1.5		21	±	1.4		95	±	0.9
		553 ng/L	8	17	↑	19,900 ± 3,570	↑	18,900 ± 5,910		97 ± 14		0 ± 0	↓	13 ± 1.9	↓	9.4	±	2.4	↓	85	±	11
	EE2	Control	0	0		0.5 ± 0.0		2,290 ± 1,040		91 ± 19		0 ± 0		23 ± 0.9		21	±	2.4		91	±	7.9
		17.8 ng/L	0	0	↑	68.3 ± 80.4		2,170 ± 223		90 ± 8.1		0 ± 0		18 ± 2.3		15	±	2.4		84	±	5.4
		84.9 ng/L	0	0	↑	10,900 ± 3,840		3,840 ± 1,370		88 ± 5.7		0 ± 0	↓	17 ± 4.9		15	±	5.0		88	±	6.1
		424 ng/L	33	8	↑	23,900 ± 3,760	↑	15,100 ± 4,290		86 ± 6.7		0 ± 0	↓	10 ± 2.8	↓	8	±	2.0	↓	73	±	3.6
	CMP	Control	0	0		0.5 ± 0.0		2,170 ± 237		100 ± 3.8		0 ± 0		29 ± 2.8		26	±	2.6		92	±	5.1
		108 μg/L	0	0		2.9 ± 4.4		2,610 ± 265		111 ± 11		0 ± 0		26 ± 3.2		24	±	3.4		94	±	5.1
		350 μg/L	0	0		9.9 ± 18.3		2,650 ± 214		109 ± 4.5		0 ± 0		25 ± 3.9		24	±	3.7		94	±	2.8
		1,060 μg/L	0	8.3	↑	33.8 ± 37.0	↑	3,020 ± 702		103 ± 6.9		0 ± 0		25 ± 2.3		23	±	2.6		93	±	2.7
	PTH	Control	0	8.3		4.7 ± 2.3		1,370 ± 243		92 ± 4.0		0 ± 0		23 ± 2.5		21	±	1.9		94	±	2.7
		96.5 μg/L	0	8.3	↑	83.2 ± 106		1,680 ± 240		98 ± 8.0		0 ± 0		19 ± 6.6		18	±	6.3		92	±	2.6
		342 μg/L	0	8.3	↑	621 ± 265		2,820 ± 1,540		94 ± 14		0 ± 0		18 ± 3.6		17	±	3.2		93	±	2.5
		1,100 μg/L	8.3	17	↑	9,740 ± 800	↑	5,770 ± 2,880		95 ± 5.0		0 ± 0	↓	13 ± 3.6	↓	10	±	4.6	↓	74	±	18
Esr antagonist	TAM	Control	0	0		6.1 ± 3.6		1,010 ± 94		96 ± 8.3		0 ± 0		22 ± 4.7		20	±	4.4		92	±	1.8
		10.0 μg/L	0	0	↑	181 ± 30.0	↓	666 ± 96		85 ± 11		0 ± 0	↓	13 ± 1.1		12	±	1.1		94	±	2.8
		28.0 μg/L	0	0	↑	226 ± 108	↓	589 ± 130		83 ± 11		0 ± 0	↓	6.0 ± 0.9	↓	5.0	±	1.3		82	±	12
		83.3 μg/L	0	8.3	↑	319 ± 126	↓	335 ± 138		85 ± 7.0		0 ± 0	↓	2.2 ± 0.8	↓	1.6	±	0.6	↓	76	±	2.0
	RAL	Control	0	0		6.7 ± 3.2		772 ± 75.6		108 ± 8.7		0 ± 0		20 ± 3.0		18	±	3.1		90	±	2.3
		63.9 μg/L	0	0		19.6 ± 5.7		581 ± 109		109 ± 12		0 ± 0		18 ± 4.2		16	±	4.6		87	±	6.6
		203 μg/L	0	0	↑	30.2 ± 11.4		652 ± 82.4		118 ± 15		0 ± 0		16 ± 2.6		14	±	2.2		86	±	4.1
		721 μg/L	0	0		24.1 ± 13.7	↓	368 ± 68.4		101 ± 5.2		0 ± 0		17 ± 2.6		14	±	2.4		82	±	4.6
		2080 μg/L	0	0		17.2 ± 3.5	↓	305 ± 99.8		103 ± 11		0 ± 0		19 ± 2.5		14	±	2.9	↓	72	±	9.1
AR agonist	MT	Control	8.3	8.3		1.5 ± 0.2		1850 ± 192		94 ± 12		0 ± 0		25 ± 2.4		23	±	2.9		94	±	2.6
		20.1 ng/L	0	0		1.5 ± 0.3		1,650 ± 134		104 ± 12	↑	28 ± 2.0		25 ± 4.6		23	±	3.8		89	±	4.0
		77.4 ng/L	0	0		2.1 ± 0.3	↓	1,080 ± 391		106 ± 7.6	↑	78 ± 7.9	↓	10 ± 2.9	↓	8.1	±	2.3		80	±	10
		300 ng/L	0	8.3		1.9 ± 0.6	↓	320 ± 266		112 ± 7.1	↑	85 ± 11	↓	1.8 ± 1.1	↓	1.4	±	0.9		79	±	14
	DHT	Control	0	0		1.4 ± 0.1		598 ± 198		61 ± 6.3		0 ± 0		22 ± 2.9		22	±	2.8		98	±	1.2
		0.063 μg/L	0	0		1.6 ± 0.4		617 ± 374		72 ± 12		0 ± 0		18 ± 5.6		17	±	5.2		94	±	1.5
		0.26 μg/L	0	0		2.3 ± 2.2		444 ± 185	↑	81 ± 7.8		2.3 ± 4.6		21 ± 2.4		20	±	2.6		97	± 1.3	
		1.03 μg/L	0	0		2.1 ± 3.0		605 ± 296		70 ± 14	↑	8.2 ± 8.1		16 ± 2.0		15	±	2.2		94	±	3.0
		2.97 μg/L	0	0		1.4 ± 0.7		587 ± 306	↑	78 ± 16	↑	22 ± 14	↓	14 ± 3.7	↓	12	±	3.7	↓	84	±	7.2
	TRB	Control	0	0		3.2 ± 1.9		685 ± 137		88 ± 4.3		0 ± 0		27 ± 3.6		26	±	4.0		95	±	2.1
		8.03 ng/L	0	0		3.7 ± 3.2		736 ± 147		86 ± 3.9		0 ± 0		27 ± 3.5		26	±	3.7		95	±	1.7
		26.8 ng/L	0	0		6.5 ± 3.8		696 ± 211		101 ± 17	↑	6.6 ± 6.1		24 ± 2.6		22	±	2.5		93	±	1.2
		84.6 ng/L	0	0		4.9 ± 4.3		658 ± 102		104 ± 8.1	↑	48 ± 6.7	↓	10 ± 2.4	↓	9.1	±	2.3		90	±	2.2
		291 ng/L	0	0		4.5 ± 2.1		641 ± 200		103 ± 8.4	↑	76 ± 3.9	↓	1.2 ± 0.5	↓	0.9	±	0.3	↓	74	±	6.3
AR antagonist	FLT	Control	0	0		38.1 ± 41.4		774 ± 163		80 ± 15		0 ± 0		29 ± 3.8		27	±	3.4		95	±	1.8
		119 μg/L	0	0		47.9 ± 41.8		908 ± 255		86 ± 18		0 ± 0		26 ± 2.3		25	±	2.7		94	±	3.8
		248 μg/L	0	0		35.8 ± 41.5		970 ± 200		82 ± 15		0 ± 0		28 ± 3.1		26	±	3.2		94	±	1.3
		497 μg/L	0	0		38.7 ± 44.8		812 ± 159		82 ± 16		0 ± 0		28 ± 3.0		27	±	3.1		96	±	1.1
		925 μg/L	0	0		50.8 ± 53.8		830 ± 131		93 ± 16		0 ± 0		29 ± 3.1		27	±	3.7		93	±	3.5
	VCZ	Control	0	0		4.5 ± 7.4		873 ± 349		100 ± 20		0 ± 0		23 ± 4.3		22	±	4.2		96	±	0.4
		14.5 μg/L	0	0		7.5 ± 7.2		886 ± 300		108 ± 18		0 ± 0		24 ± 2.6		22	±	2.3		95	±	1.3
		43.9 μg/L	0	0		10 ± 12		816 ± 278		95 ± 16		0 ± 0		23 ± 5.3		22	±	4.9		94	±	3.2
		137 μg/L	0	0		3.6 ± 2.3		651 ± 217		108 ± 16		0 ± 0		21 ± 4.3		19	±	4.6		90	±	3.9
		453 μg/L	0	0		3.9 ± 8.6	↓	111 ± 92		102 ± 17		0 ± 0		20 ± 5.9		17	±	3.9	↓	84	±	10
Steroidogenesis Inhibitor	KCZ	Control	0	0		3.0 ± 3.1		812 ± 192		79 ± 18		0 ± 0		22 ± 2.3		19	±	3.4		90	±	11
		105 μg/L	0	0	↓	1.4 ± 0.3		618 ± 81		82 ± 10		0 ± 0		24 ± 1.1		22	±	2.3		91	±	3.8
		233 μg/L	0	0	↓	1.3 ± 0.1	↓	542 ± 353	↑	116 ± 25		0 ± 0		20 ± 1.6		18	±	4.9		89	±	4.4
		405 μg/L	0	8	↓	1.1 ± 0.1	↓	190 ± 123		91 ± 13		0 ± 0	↓	11 ± 4.4	↓	9.0	±	4.6		81	±	3.9
		795 μg/L	58	42	↓	1.1 ± 0.1	↓	56 ± 95		80 ± 22		0 ± 0	↓	4.7 ± 0.9	↓	3.5	±	3.9	↓	76	±	5.5
	PCL	Control	8.3	0		15.4 ± 27.4		428 ± 350		104 ± 15		0 ± 0		34 ± 2.6		32	±	2.7		96	±	3.3
		10.3 μg/L	8.3	0		6.7 ± 7.4		301 ± 52		99 ± 13		0 ± 0		39 ± 4.3		38	±	4.4		95	±	3.1
		20.6 μg/L	0	0		2.6 ± 2.0		289 ± 57		103 ± 15		0 ± 0		39 ± 4.5		37	±	4.6		94	±	3.4
		44.9 μg/L	0	0		1.2 ± 1.2	↓	173 ± 53		93 ± 13		0 ± 0		34 ± 5.5		28	±	6.3	↓	84	±	10
PR agonist	P4	Control	0	0		32 ± 25		934 ± 447		102 ± 12		0 ± 0		21 ± 2.6		20	±	2.6		95	±	7.1
		11 μg/L	0	0		33 ± 29		746 ± 419		109 ± 14	↑	11 ± 8.9		18 ± 3.8	↓	17	±	3.8		92	±	3.0
		24 μg/L	0	8		29 ± 22		818 ± 270		108 ± 20	↑	21 ± 5.9	↓	17 ± 4.0	↓	16	±	4.0	↓	91	±	7.8
		64 μg/L	0	8		37 ± 27		1,008 ± 445		101 ± 13	↑	35 ± 8.9	↓	16 ± 6.5	↓	15	±	6.4	↓	87	± 7.2	
		243 μg/L	50	83		37 ± 36		899 ± 139		100 ± 10	↑	32 ± 21	↓	1.0 ± 2.3	↓	0.2	±	0.8	↓	2.9	±	6.8
	LNG	Control	0	8		1.00 ± 0.4		2,808 ± 522		114 ± 7.5		0 ± 0		30 ± 4.9		28	±	4.5		92	±	2.3
		7.3 ng/L	0	0		2.8 ± 4.1		2,370 ± 207		112 ± 4.5		0 ± 0		29 ± 5.8		25	±	6.1		87	±	7.2
		42.2 ng/L	0	8		0.8 ± 0.3		2,350 ± 673		121 ± 8.6	↑	44 ± 10		21 ± 3.9	↓	17	±	4.3		82	±	8.1
		226 ng/L	0	0		0.6 ± 0.1	↓	1,120 ± 561		116 ± 10	↑	83 ± 5.4	↓	5.9 ± 5.2	↓	5.1	±	4.7		82	±	10
Negative	SDS	Control	0	0		1.8 ± 1.6		4,440 ± 237		109 ± 8.2		0 ± 0		28 ± 2.1		26	±	2.9		91	±	4.1
		0.748 mg/L	0	0		3.0 ± 4.2	↓	3,780 ± 305		107 ± 8.7		0 ± 0		29 ± 3.9		27	±	4.3		95	±	2.2
		2.46 mg/L	0	0		6.6 ± 11	↑	4,930 ± 189		112 ± 11		0 ± 0		30 ± 2.7		27	±	2.1		93	±	3.6
		10.0 mg/L	0	0		1.0 ± 0.5		4,520 ± 906		110 ± 3.5		0 ± 0		30 ± 1.8		25	±	1.9	↓	85	±	2.2

*Note*. Data denoted in mean ± standard deviation and arrows indicate that a significant increase (↑) or decrease (↓) from the control was found (*p* < 0.05).

Abbreviations: CMP, 4‐chloro‐3‐methylphenol; DHT, 5*α*‐dihydrotestosterone; E2, 17*β*‐estradiol; EE2, 17*α*‐ethynylestradiol; FLT, flutamide; FSTRA, fish short‐term reproduction assay; KCZ, ketoconazole; LNG, levonorgestrel; MT, 5*α*‐methyltestosterone; P4, progesterone; PCL, prochloraz; PTH, 4‐*tert*‐pentylphenol; RAL, raloxifene hydrochloride; SDS, sodium dodecyl sulfate; SSC, secondary sex characteristics (number of joint plates with papillary processes on anal fin); TAM, tamoxifen citrate; TRB, 17*β*‐trenbolone; VCZ, vinclozolin; VTG, hepatic vitellogenin concentration.

**TABLE 6:** Length, weight, HSI, and GSI at the completion of the exposure in FSTRAs

Test chemical		Measured	Length (mm)						Weight (mg)						HSI (%)						GSI (%)					
		Conc.	Male			Female			Male			Female			Male			Female			Male			Female		
Esr agonist	E2	Control	35.5	±	1.1	35.5	±	1.5	476	±	60	535	±	80	2.1	±	0.40	4.0	±	0.80	0.74	±	0.16	9.8	±	1.1
		22.1 ng/L	36.4	±	1.3	36.8	±	1.7	507	±	73	573	±	67	2.0	±	0.39	4.4	±	0.71	0.82	±	0.17	10.3	±	1.7
		115 ng/L	35.8	±	1.2	35.6	±	1.9	512	±	49	517	±	60	2.5	±	0.40	4.3	±	0.63	0.67	±	0.18	11.4	±	3.1
		553 ng/L	35.0	±	1.4	35.0	±	1.3	488	±	83	512	±	73	3.2	±	0.82	4.4	±	0.90	0.31	±	0.12	11.2	±	7.7
	EE2	Control	36.5	±	1.5	36.2	±	1.8	567	±	71	523	±	65	2.5	±	0.64	3.8	±	0.72	0.62	±	0.27	9.6	±	1.1
		17.8 ng/L	35.9	±	2.2	35.4	±	0.9	531	±	104	516	±	56	2.3	±	0.32	4.2	±	0.68	0.75	±	0.36	10.0	±	0.9
		84.9 ng/L	36.3	±	1.2	35.8	±	1.2	548	±	59	498	±	53	2.7	±	0.65	3.7	±	0.64	0.60	±	0.19	9.5	±	1.8
		424 ng/L	35.1	±	0.9	35.3	±	1.1	531	±	104	507	±	52	3.4	±	0.53	3.8	±	0.72	0.33	±	0.17	10.4	±	5.4
	CMP	Control	36.5	±	1.7	35.3	±	1.1	498	±	85	526	±	56	1.6	±	0.40	4.1	±	0.61	0.73	±	0.19	9.4	±	1.3
		108 μg/L	36.0	±	1.3	36.5	±	1.8	498	±	64	565	±	66	1.9	±	0.34	3.9	±	0.74	0.80	±	0.41	9.9	±	1.3
		350 μg/L	36.4	±	1.5	35.4	±	1.3	529	±	76	509	±	69	2.4	±	0.83	4.4	±	0.57	0.68	±	0.11	9.8	±	1.0
		1,060 μg/L	36.3	±	1.4	35.2	±	1.2	505	±	89	509	±	65	2.0	±	0.34	3.9	±	0.65	0.74	±	0.21	9.9	±	1.1
	PTH	Control	35.8	±	1.1	34.2	±	1.8	493	±	97	482	±	71	2.8	±	0.94	4.5	±	0.85	0.76	±	0.20	9.8	±	1.7
		96.5 μg/L	35.8	±	2.1	34.8	±	1.6	532	±	91	470	±	61	2.5	±	0.87	4.3	±	0.81	0.74	±	0.31	9.5	±	1.4
		342 μg/L	34.7	±	1.1	34.5	±	1.2	516	±	57	476	±	49	3.1	±	0.52	4.5	±	0.46	0.67	±	0.18	8.1	±	1.6
		1,100 μg/L	34.5	±	1.6	33.3	±	1.0	498	±	115	438	±	39	4.1	±	0.77	4.7	±	0.70	0.51	±	0.12	8.4	±	1.6
Esr antagonist	TAM	Control	34.2	±	1.5	33.8	±	1.0	443	±	53	461	±	48	2.2	±	0.34	4.9	±	0.78	0.68	±	0.20	9.6	±	1.8
		10.0 μg/L	33.6	±	1.9	32.3	±	1.4	419	±	87	369	±	43	2.2	±	0.47	3.6	±	0.72	0.89	±	0.16	7.3	±	2.1
		28.0 μg/L	33.1	±	1.5	33.3	±	2.0	396	±	49	432	±	93	2.1	±	0.23	3.9	±	0.44	0.74	±	0.16	9.7	±	2.7
		83.3 μg/L	32.5	±	0.7	32.9	±	1.4	374	±	31	418	±	80	2.5	±	0.45	4.2	±	0.85	0.87	±	0.18	11.5	±	5.7
	RAL	Control	37.6	±	0.1	36.8	±	0.3	532	±	25	555	±	9.2	1.4	±	0.31	2.5	±	0.48	1.00	±	0.08	9.1	±	0.5
		63.9 μg/L	37.5	±	0.9	36.9	±	0.7	511	±	45	555	±	37	1.5	±	0.06	3.2	±	0.44	1.03	±	0.11	9.6	±	1.1
		203 μg/L	38.4	±	1.5	37.8	±	0.6	550	±	62	600	±	14	1.6	±	0.23	2.9	±	0.77	0.97	±	0.03	10.2	±	1.6
		721 μg/L	39.0	±	1.1	38.8	±	0.5	612	±	68	638	±	30	1.5	±	0.09	3.4	±	0.07	0.96	±	0.19	10.5	±	0.9
		2080 μg/L	37.1	±	1.4	38.8	±	0.5	506	±	73	675	±	25	1.7	±	0.38	4.7	±	0.31	1.12	±	0.12	11.5	±	1.4
AR agonist	MT	Control	37.1	±	1.7	36.2	±	1.5	562	±	70	596	±	63	2.6	±	0.41	4.5	±	0.89	0.81	±	0.25	9.6	±	0.9
		20.1 ng/L	37.3	±	1.7	36.1	±	1.6	546	±	89	592	±	66	2.5	±	0.74	4.8	±	0.88	0.68	±	0.15	13.5	±	1.8
		77.4 ng/L	38.3	±	1.3	36.7	±	1.6	603	±	64	694	±	88	2.4	±	0.51	4.4	±	0.55	0.78	±	0.12	18.6	±	2.7
		300 ng/L	37.5	±	1.5	37.0	±	1.8	561	±	76	654	±	68	2.5	±	0.48	4.2	±	0.90	0.68	±	0.22	12.6	±	5.4
	DHT	Control	32.9	±	1.4	33.0	±	1.6	352	±	61	368	±	61	1.7	±	0.48	5.2	±	1.17	1.44	±	1.01	10.8	±	1.9
		0.063 μg/L	33.4	±	2.2	32.9	±	1.5	377	±	92	354	±	63	1.5	±	0.48	3.6	±	0.92	1.15	±	0.27	10.5	±	1.9
		0.26 μg/L	33.5	±	1.8	32.9	±	1.8	389	±	59	343	±	51	1.8	±	0.31	4.4	±	1.05	1.04	±	0.15	12.6	±	2.1
		1.03 μg/L	32.8	±	1.4	32.6	±	1.4	389	±	71	356	±	69	1.6	±	0.33	2.9	±	0.68	1.11	±	0.14	15.5	± 2.4	
		2.97 μg/L	33.6	±	1.7	32.8	±	1.6	411	±	110	425	±	121	1.7	±	0.31	3.8	±	0.52	1.21	±	0.28	21.1	±	2.8
	TRB	Control	36.1	±	1.1	35.3	±	0.4	501	±	51	549	±	20	1.9	±	0.15	5.0	±	0.30	1.05	±	0.14	10.8	±	1.0
		8.03 ng/L	36.7	±	1.3	36.4	±	2.0	502	±	60	573	±	74	1.8	±	0.17	5.4	±	0.52	1.04	±	0.15	9.7	±	0.3
		26.8 ng/L	37.3	±	1.1	35.6	±	1.1	500	±	64	515	±	39	2.1	±	0.19	5.5	±	0.25	1.06	±	0.14	11.3	±	0.7
		84.6 ng/L	37.7	±	1.6	36.1	±	0.8	524	±	51	625	±	61	2.1	±	0.37	3.7	±	0.86	1.11	±	0.09	16.1	±	1.3
		291 ng/L	37.0	±	0.5	37.3	±	0.4	504	±	29	691	±	29	1.8	±	0.19	3.4	±	0.35	1.10	±	0.07	18.8	±	1.9
AR antagonist	FLT	Control	34.6	±	1.0	33.2	±	0.8	414	±	64	389	±	32	1.9	±	0.33	4.6	±	0.65	0.90	±	0.32	9.2	±	1.0
		119 μg/L	35.6	±	0.9	34.2	±	1.7	445	±	48	426	±	76	2.0	±	0.63	4.2	±	0.65	1.10	±	0.26	10.1	±	1.8
		248 μg/L	35.0	±	1.7	33.7	±	1.2	394	±	60	428	±	50	2.1	±	0.44	4.6	±	0.63	1.10	±	0.19	10.9	±	2.8
		497 μg/L	35.9	±	1.8	34.3	±	1.4	429	±	73	470	±	51	1.9	±	0.35	4.8	±	0.87	1.20	±	0.28	10.3	±	1.0
		925 μg/L	35.8	±	1.2	34.2	±	1.3	416	±	42	453	±	58	2.0	±	0.38	4.5	±	1.41	1.10	±	0.23	11.5	±	0.9
	VCZ	Control	34.2	±	1.8	33.8	±	0.9	411	±	63	420	±	41	2.0	±	0.44	4.2	±	1.44	1.15	±	0.27	9.9	±	1.8
		14.5 μg/L	34.8	±	1.5	35.2	±	1.2	455	±	74	447	±	54	1.7	±	0.50	4.6	±	0.98	1.09	±	0.28	11.8	±	1.7
		43.9 μg/L	34.4	±	1.4	35.0	±	1.5	427	±	80	445	±	69	1.7	±	0.38	4.7	±	0.85	1.24	±	0.16	10.7	±	0.7
		137 μg/L	34.4	±	2.0	34.8	±	1.9	425	±	75	437	±	77	1.5	±	0.43	4.5	±	1.03	1.40	±	0.39	10.6	±	1.6
		453 μg/L	34.9	±	1.5	34.8	±	1.9	455	±	94	455	±	122	1.8	±	0.23	4.7	±	1.07	1.46	±	0.28	12.0	±	1.3
Steroidogenesis Inhibitor	KCZ	Control	33.0	±	1.7	33.8	±	1.7	352	±	65	378	±	66	1.6	±	0.52	4.2	±	0.88	1.52	±	0.99	9.6	±	0.9
		105 μg/L	32.7	±	1.2	33.2	±	1.0	381	±	45	387	±	54	1.9	±	0.72	4.2	±	0.96	1.08	±	0.31	11.6	±	1.7
		233 μg/L	33.3	±	1.5	33.3	±	1.3	416	±	79	419	±	78	3.8	±	1.35	8.8	±	2.24	1.26	±	0.45	12.0	±	2.7
		405 μg/L	32.4	±	1.5	32.6	±	1.0	404	±	86	410	±	68	3.5	±	1.30	12.1	±	2.88	0.98	±	0.25	13.6	±	4.2
		795 μg/L	30.2	±	1.4	31.7	±	1.0	282	±	64	376	±	59	5.1	±	2.59	11.8	±	4.05	1.35	±	0.44	11.0	±	3.5
	PCL	Control	34.3	±	1.2	35.4	±	1.7	400	±	53	535	±	103	1.5	±	0.36	4.6	±	1.07	1.34	±	0.24	11.3	±	2.4
		10.3 μg/L	34.4	±	1.8	36.0	±	1.1	414	±	68	523	±	64	1.5	±	0.31	4.2	±	0.49	1.09	±	0.25	9.3	±	1.2
		20.6 μg/L	34.4	±	1.5	35.0	±	2.8	408	±	58	547	±	72	1.7	±	0.30	4.2	±	1.27	1.26	±	0.26	10.2	±	1.0
		44.9 μg/L	34.3	±	1.9	36.5	±	1.6	415	±	76	591	±	97	1.7	±	0.45	4.5	±	1.51	1.16	±	0.26	11.1	±	0.9
PR agonist	P4	Control	36.8	±	1.3	38.6	±	1.1	482	±	62	645	±	122	1.9	±	0.20	5.7	±	2.01	1.19	±	0.27	14.8	±	5.3
		11 μg/L	36.9	±	0.9	37.8	±	0.9	501	±	44	647	±	59	2.0	±	0.32	5.2	±	0.69	1.20	±	0.14	13.4	±	1.2
		24 μg/L	36.4	±	1.4	38.0	±	1.2	475	±	64	660	±	85	1.9	±	0.29	4.8	±	0.65	1.04	±	0.15	14.1	±	2.3
		64 μg/L	37.1	±	1.4	38.2	±	1.7	465	±	69	696	±	112	2.8	±	0.57	4.9	±	0.91	1.14	±	0.25	13.9	±	1.0
		243 μg/L	36.9	±	1.1	35.3	±	2.2	479	±	49	804	±	411	2.1	±	0.60	3.2	±	0.59	1.15	±	0.23	36.9	±	13.2
	LNG	Control	39.2	±	1.4	37.2	±	1.4	658	±	100	600	±	43	2.2	±	0.45	4.0	±	0.86	0.85	±	0.24	9.9	± 0.8	
		7.3 ng/L	38.4	±	0.9	37.8	±	1.1	610	±	72	622	±	42	2.0	±	0.43	4.0	±	0.26	0.82	±	0.14	11.0	±	1.7
		42.2 ng/L	38.8	±	1.9	38.5	±	1.1	691	±	137	731	±	70	2.2	±	0.40	3.5	±	0.63	0.89	±	0.18	17.9	±	4.0
		226 ng/L	38.3	±	1.3	38.7	±	1.2	610	±	91	788	±	70	2.1	±	0.30	3.3	±	0.69	0.76	±	0.24	17.0	±	2.8
Negative	SDS	Control	36.8	±	1.7	36.1	±	1.6	525	±	79	557	±	82	1.5	±	0.73	4.0	±	0.74	0.84	±	0.17	10.1	±	1.0
		0.748 mg/L	36.7	±	1.9	35.6	±	1.1	544	±	100	547	±	52	2.3	±	0.31	4.8	±	0.53	0.88	±	0.25	10.9	±	0.9
		2.46 mg/L	36.7	±	1.3	36.6	±	1.3	519	±	52	573	±	59	2.0	±	0.29	4.8	±	0.49	0.91	±	0.27	10.8	±	1.4
		10.0 mg/L	35.6	±	1.2	36.1	±	1.4	493	±	57	573	±	56	2.2	±	0.26	4.9	±	0.69	1.17	±	0.40	11.0	±	1.3

Abbreviations: AR, androgen receptor; CMP, 4‐chloro‐3‐methylphenol; DHT, 5*α*‐dihydrotestosterone; E2, 17*β*‐estradiol; EE2, 17*α*‐ethynylestradiol; Esr, estrogen receptor; FLT, flutamide; FSTRA, fish short‐term reproduction assay; GSI, gonadosomatic index; HSI, hepatosomatic index; KCZ, ketoconazole; LNG, levonorgestrel; MT, 5*α*‐methyltestosterone; P4, progesterone; PCL, prochloraz; PTH, 4‐*tert*‐pentylphenol; RAL, raloxifene hydrochloride; SDS, sodium dodecyl sulfate; TAM, tamoxifen citrate; TRB, 17*β*‐trenbolone; VCZ, vinclozolin.

For the mortalities in controls, the test validity criterion suggested in the OECD TG229, less than 10%, was satisfied in all the studies (Table 5). In the chemical treatments, a remarkable high mortality was found in males (33%) at the highest concentration of EE2, in both males (59%) and females (42%) for KCZ, and in both males (50%) and females (83%) in P4 exposure. The highest concentrations of E2 and PTH caused a slight increase in mortality (17%).

#### 3.1.3 Reproduction (spawning status)

##### Number of total eggs

In the controls, the fecundity that is denoted as an average number of total eggs one female medaka laid a day during the 21‐day exposure period ranged between 19 and 34 eggs over the 16 studies, as shown in Table 5. Among the Esr agonists, a significant decrease was observed in the fecundity for E2, EE2, and PTH studies, but an overt toxicity, that is, increased mortality, was also found at the same concentrations, except at the middle concentration of E2. In the Esr antagonist studies, a concentration‐dependent and a statistically significant decrease was found in all three concentrations for TAM. All the three AR agonists, MT, TRB and DHT, significantly reduced the number of spawned eggs, compared with the controls, at the concentration in which no overt toxicity was observed. A concentration‐dependent decrease and a significant difference from the control at the concentrations in which the mortality was less than 10% was observed in both the PR agonists (P4 and LNG) and the KCZ studies. No effect on fecundity within the test concentrations was suggested in the fish exposed to CMP, FLT, VCZ, PCL, and SDS (Table 5).

##### Number of fertile eggs

In the 12 studies other than those with EE2, TAM, P4, and LNG, the LOECs on number of fertile eggs were the same as the LOECs on fecundity (number of total eggs). In the EE2 and the TAM studies, the LOECs on number of fertilized eggs were one concentration higher, which is less sensitive, than that for the total eggs, and conversely, was one concentration lower in the two PR agonist studies (Table 5).

##### Fertility rate

The mean of the fertility rate during the 21‐day exposure period in the controls ranged between 90% and 98% over the 16 studies. A significant decrease could be detected for four chemicals (RAL, VCZ, PCL, and SDS) in which no effect on both the number of total and fertilized eggs was detected (Table 5).

#### 3.1.4 Hepatic VTG

VTG induction is generally at a low level in male fish and hence has been widely used as a biomarker to screen chemicals for estrogenic activity on fish over the years (Hansen et al., 1998; Kime, Nash, & Scott, 1999; OECD, 2009a; Sumpter & Jobling, 1995; Tyler, van der Eerden, Jobling, Panter, & Sumpter, 1996). In the present study, the mean hepatic VTG levels in the control males were less than 10 ng/mg of liver, except for the FLT, PCL, and P4 studies, where the mean values for VTG in control males increased up to a maximum of 38 ng/mg of liver. As to the mean of VTG levels in control females over the 16 studies, a variation between 428 and 4,440 ng/mg of liver, which might be depended on the types or interlot variation of the ELISA kits used, was recognized (Table 5).

In the Esr agonist studies, the male VTG levels were significantly elevated depending on the exposure concentrations and exceeded the VTG levels in the control females at the highest concentrations for E2, EE2, and PTH. The LOECs for VTG induction in males were 22.1 ng/L, 17.8 ng/L, 1,060μg/L, and 96.5 μg/L for E2, EE2, CMP, and PTH, respectively. With regard to the female hepatic VTG, a significant increase was found in all the four Esr agonist studies, but the LOECs were higher than the LOECs on male VTG in all the studies, except for CMP. An increase of VTG in male medaka was also caused by the Esr antagonist exposure, but a concentration dependence was not obvious in the RAL study. On the other hand, VTG levels in females were concentration dependently decreased by both the TAM and the RAL treatment, and the LOECs were respectively 10.0 and 721 μg/L. Among the AR agonists, only MT decreased female VTG levels in a concentration‐dependent manner and a significant difference from the control could be detected in the two highest concentrations. KCZ and PCL, steroidogenesis inhibitors, reduced hepatic VTG in females and the LOEC in which no mortality was caused was suggested to be 233 and 44.9 μg/L, respectively. The AR antagonists altered the hepatic VTG levels in neither males nor females. Regarding the PR agonists, female hepatic VTG was significantly decreased at the highest concentration of 226 ng/L in the LNG treatment, but P4 caused no alteration at any exposure concentration. In the SDS study, an increase and a decrease, which were statistically significant but suspected as not being caused by interaction with the HPG axis, were observed in females but no VTG induction was found in males.

#### 3.1.5 Secondary sex characteristics

The formation of PPs on anal fin rays is masculine SSC in Japanese medaka. The knowledge that the development of PP is promoted by androgen‐dependent augmentation of bone morphogenic protein 7 and lymphoid enhancer‐binding factor‐1 in males and can be induced in females by exogenous androgen exposure, that is, exposing to AR agonistic chemicals, has been reported by Ogino et al. (2014). In the controls over the 16 chemical studies, the mean values of the SSC, that is, the number of joint plates with PP on anal fin rays, ranged from approximately 60 to 120 in males, whereas none were consistently observed in females.

The three AR agonists, MT, DHT, and TRB, and both the PR agonists, P4 and LNG, induced PP on the anal fin in female medaka, and the count values of SSC were concentration‐dependently increased in all the five chemical exposures. The LOECs on female for MT, DHT, TRB, P4, and LNG were 20.1 ng/L, 1.03 μg/L, 26.8 ng/L, 11 μg/L and 42.2 ng/L, respectively, and were lower than the LOECs on the other endpoints. In the AR agonist studies, a tendency for SSC to be slightly increased in a concentration‐dependent manner was also found in males, but a statistical significance from the control was detected only in the DHT study. Regarding the other MOAs including AR antagonists, no alteration in either male or female SSC was observed, although a statistical significance, which might be caused by a factor not associated with the MOA of the test chemical, was found in the KCZ study (Table 5).

### 3.2 Medaka Esr1 and AR*β* RGAs

The results of RGAs, the agonist and antagonist assays using mEsr1 and mAR*β*, are summarized in Table 7. Regarding the agonist assays using mEsr1, EC_50_ of a positive control E2, to confirm the verification of the assay, was 0.00098 μM (9.8 × 10^−10^ M), and EC_50_s of 0.00088, 0.97, and 61 μM were obtained for EE2, PTH, and CMP, respectively. Conversely, an EC_50_ could not be determined for the negative substance SDS because a significant increase in hold activation was not shown even at the highest concentration of 100 μM. In addition, the two PR agonists were assayed in the mEsr1 agonist assay, and an EC_50_ of 1.1 μM was obtained only for LNG. In the antagonist assays with mEsr1, IC_50_s of 0.14, 0.0026, and 0.00052 μM were obtained for TAM, RAL, and 4HTAM (a positive control), respectively, while no Esr inhibiting activity in the 1 nM E2‐mediated mEsr1 transactivation was observed for all the test chemicals of AR agonists, steroidogenesis inhibitors, PR agonists, and the negative substance SDS.

**TABLE 7:** Results of agonist and antagonist assays for mEsr1 and mAR*β* RGA

Test chemical		Agonist assay, EC50 (μM)		Antagonist assay, IC50 (μM)	
		Medaka Esr1	Medaka AR*β*	Medaka Esr1	Medaka AR*β*
Esr agonist	E2^a^	0.00098	na	na	2.0
	EE2	0.00088	na	na	0.14
	CMP	61	na	na	82
	PTH	0.97	na	na	ND (>3.2)
Esr antagonist	4HTAM^a^	na	na	0.00052	na
	TAM	ND (>10)	na	0.14	na
	RAL	ND (>10)	na	0.0026	na
AR agonist	11KT^a^	na	0.0027	ND (>100)	na
	MT	na	0.00012	ND (>10)	na
	DHT	na	0.49	ND (>10)	na
	TRB	na	0.0036	ND (>10)	na
AR antagonist	2HFLT^a^	na	na	na	0.33
	FLT	na	ND (>10)	na	12
	VCZ	na	ND (>100)	na	5.1
Steroidogenesis inhibitor	KCZ	na	na	ND (>10)	4.2
	PCL	na	na	ND (>10)	ND (>10)
PR agonist	P4	ND (>10)	9.1	ND (>10)	na
	LNG	1.1	0.000013	ND (>100)	na
Negative	SDS	ND (>100)	ND (>100)	ND (>100)	ND (>100)

Abbreviation: 11KT, 11‐ketotestosterone; 2HFLT, 2‐hydroxy flutamide; 4HTAM, 4‐hydroxy tamoxifen; AR, androgen receptor; CMP, 4‐chloro‐3‐methylphenol; DHT, 5*α*‐dihydrotestosterone; E2, 17*β*‐estradiol; EE2, 17*α*‐ethynylestradiol; Esr, estrogen receptor; FLT, flutamide; KCZ, ketoconazole; LNG, levonorgestrel; MT, 5*α*‐methyltestosterone; na, not available data; ND, EC_50_ or IC_50_ could not be determined because a significant response was not shown even at the highest assay concentration expressed in parentheses; P4, progesterone; PCL, prochloraz; PTH, 4‐*tert*‐pentylphenol; RAL, raloxifene hydrochloride; RGA, Esr, estrogen receptor; SDS, sodium dodecyl sulfate; TAM, tamoxifen citrate; TRB, 17*β*‐trenbolone; VCZ, vinclozolin.

^a^ E2, 4HTAM, 11KT, and 2HFLT were used as a positive control substance to confirm that the assay appropriately worked for Esr agonist, Esr antagonist, AR agonist, and AR antagonist assay, respectively.

In the agonist assays using mAR*β*, the EC_50_ of 11KT for the positive control was 0.0027 μM and for MT, DHT, and TRB was 0.00012, 0.49, and 0.0036 μM, respectively. The two PR agonists also indicated a positive response, and in particular the EC_50_ for LNG was 0.000013 μM, indicating that it was 50 times more potent than the positive control 11KT. Again, no significant increase in hold activation was found for SDS. With regard to the antagonist assays, IC_50_ on 50 nM 11KT‐induced mAR*β* agonistic activity could be determined for three Esr agonists (E2, EE2, and CMP) and the steroidogenesis inhibitor KCZ, as well as for the AR antagonists including the positive control 4HFLT. The IC_50_ for the AR antagonistic effect was 0.33, 12, and 5.1 μM for 2HFLT, FLT, and VCZ, respectively, whereas EE2 had the lowest IC_50_ of 0.14 μM.

## 4 DISCUSSION

In the FSTRA using Japanese medaka, mature adult males and females were exposed together to each test chemical in a test vessel for 3 weeks. The chemical treatment was conducted with a minimum of three concentrations and an appropriate control in which four replicate tanks to ensure an adequate statistical power in analysis of endpoint data were containing. As apical endpoints to evaluate endocrine disrupting effects of the test chemicals, the eggs females produced were daily counted separately for fertilized and unfertilized in each tank during the exposure period, and the hepatic VTG and the SSC (i.e., the number of joint plates on anal fin rays which PPs formed) were quantitatively measured in all the fish survived at the completion of the exposure.

**FIGURE 1:**
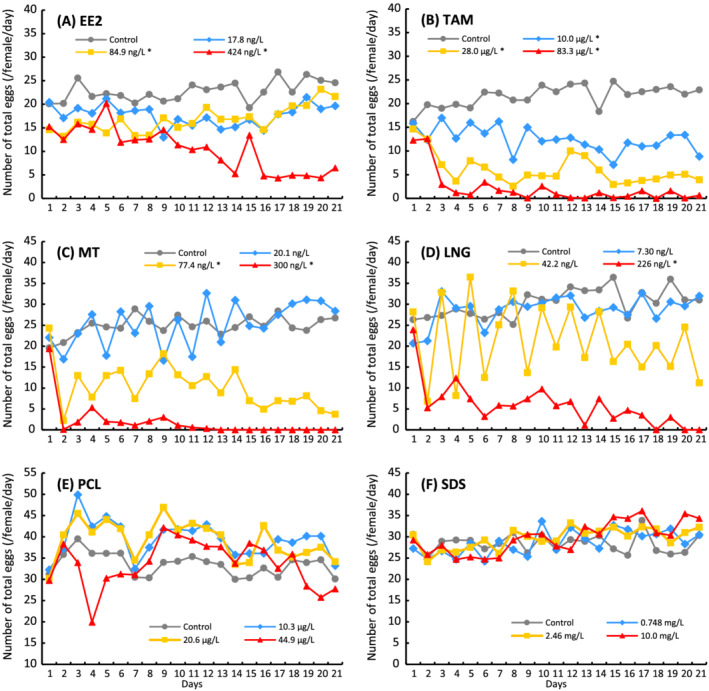
Daily change in the mean number of total eggs during the 21‐day exposure period. Data denote the mean of the daily fecundity (*n* = 4). The exposure concentrations in which a significant reduction from the control was statistically detected in the number of total eggs throughout the exposure period were marked with an asterisk (*p* < 0.05). (A) 17*α*‐Ethynylestradiol, (B) tamoxifen citrate, (C) 5*α*‐methyltestosterone, (D) levonorgestrel, (E) prochloraz, and (F) sodium dodecyl sulfate

Fecundity and fertility can be the most useful indicators of the general reproductive condition of mature fish because these endpoints reflect the successful integration of a variety of physiological processes, for example, disturbances in the HPG axis that directly or indirectly impair gamete maturation and/or interfere with reproductive behavior will reduce spawning frequency and fecundity (US EPA, 2007). In the FSTRA (TG229) guideline, on the other hand, it is also suggested that these endpoints are not intended to unequivocally identify specific cellular mechanisms of action (OECD, 2012a). In the present study, a significant reduction in both the number of total and fertilized eggs was able to statistically be detected for the 10 chemicals other than CMP, RAL, FLT, VCZ, PCL, and SDS (Table 5). These results demonstrated that various MOA, except AR antagonism, that might interact with the HPG axis mostly affect the reproduction of mature medaka and also suggested that it is probably difficult to determine the MOA of a test chemical based on the changes in the average egg production (/day/female) during the exposure period. In the FSTRA, because the eggs spawned in each tank must be recorded every day, the time course of fecundity and fertility over the exposure period can be assessed. As shown in Figure 1, different trends (variation patterns) supposed to be associated with the MOA of the test chemicals were found in the daily egg production over the exposure period. The fecundity of the fish exposed to Esr agonist at higher concentration (e.g., EE2 at 424 ng/L) gradually decreased after the first week (Figure 1A). In contrast, the exposure to TAM, an Esr antagonist, drastically reduced the daily fecundity within 3 days from the initiation of chemical treatment (Figure 1B). Similarly, the egg production dropped more rapidly on the day after the beginning of the exposure with strong AR agonist treatment, but unlike the TAM treatment, large fluctuations in daily fecundity were found in the MT exposure, especially at the lowest concentration (Figure 1C). A similar response was evident in the fish exposed to PR agonists such as LNG (Figure 1D). Interestingly, in the PCL study where a significant reduction could not statistically be detected in the mean number of total eggs during the 21 days, the daily fecundity was rather reduced a few days from the start of the chemical exposure at the highest concentration but recovered after that (Figure 1E).

The daily fecundity in controls that was the average of the number of total eggs spawned by 12 females must be stable over the exposure period where the feeding and the environmental conditions are properly controlled under the FSTRA protocol because Japanese medaka is a daily spawner (Ankley & Johnson, 2004; Leaf et al., 2011; Padilla et al., 2009). It is assumed that the variation in daily fecundities caused by the chemical treatments reflected MOA of the chemicals tested and reproductive response of the fish exposed, although the fecundity endpoint in the FSTRA might not be sensitive to AR antagonistic effects (Ankley et al., 2003; Dang, Traas, & Vermeire, 2011; Nakamura et al., 2014). The results from the EE2 study can be interpreted as that the strong Esr agonist interfered with certain functions of male medaka, for example, spermatogenesis and reproductive behavior (Islinger, Willimski, Völkl, & Braunbeck, 2003; Schultz, Skillman, Nicolas, Cyr, & Nagler, 2003; Seki et al., 2002), and then reduced the reproductive activity of females in the same tank, as a secondary effect. A significant decreasing male GSI at the highest concentrations of E2, EE2, and PTH, shown in Table 6, supports this interpretation. Several literatures have reported that a short‐term exposure of adult males and females to synthetic androgens such as MT and TRB caused a reduction of egg production in medaka, fathead minnow, and other small test fish (Ankley et al., 2003; Jensen, Makynen, Kahl, & Ankley, 2006; Kang et al., 2008; Pawlowski, Sauer, Shears, Tyler, & Braunbeck, 2004; Robinson, Staveley, & Constantine, 2017). In the present study, a large fluctuation was observed in daily egg production in addition to the decrease in total egg production in the treatment by AR agonists. These results suggest that androgen treatment might not only interfere with oogenesis but also disrupt maturational and ovulatory mechanisms resulting in inhibition of the normal release of mature oocytes during spawning (Hemmer et al., 2008). Consequently, in the females treated with the AR agonists, the GSI was probably elevated by the increase of matured oocytes retained in their ovaries. As shown in Table 6, an increased GSI in female was also evident in the exposure with PR agonists. Several in vivo studies have suggested that synthetic progestins including LNG significantly affected transcriptional expression levels of genes related to HPG axis and reduced reproductive capability in fish (Frankel, Meyer, & Orlando, 2016; Han et al., 2014; Orlando & Ellestad, 2014; Paulos et al., 2010; Runnalls et al., 2013; Svensson, Fick, Brandt, & Brunström, 2013; Zeilinger et al., 2009). The in vitro RGA with mAR*β* demonstrated that the natural progesterone P4 and the synthetic progestin LNG were potent AR agonists in Japanese medaka, as shown in Table 7, the same as in other fish species such as fathead minnow (Bain, Kumar, Ogino, & Iguchi, 2015; Ellestad et al., 2014). In the SDS study, used as a negative substance, no significant effect compared with the control was found for the number of total eggs, but a significant reduction was statistically detected in the fertility rate at the highest concentration (Table 5). With regard to the fertility rate, a significant reduction compared with the control was also detected in the studies for RAL, VCZ, and PCL where no effect was statistically detected in both the number of total and fertile eggs. These results suggest that fertility rate is more sensitive than the other two endpoints for reproduction in FSTRA. Part of the reason for this is that the variations of fertility rates in the controls were relatively smaller than the other endpoints. At the same time, it should be considered a possibility that some toxic effect unrelated to endocrine disruption caused the reduction in fertility. For example, in the 10 mg/L of SDS exposure, it is presumed that surfactant lipid peroxidation of SDS damaged the sperms and this consequently reduced fertility (Dietrich et al., 2007; Rosety et al., 2001, 2007). The results of RGA showed that SDS has neithEsr agonistic nor antagonistic activity to both mAR*β* and mEsr1, supporting this interpretation. Overall, the 16 chemical studies demonstrated that the reproductive endpoints in the FSTRA using Japanese medaka could be fairly sensitive and helpful to detect the effects of test chemicals on HPG axis, although it would be necessary to suspect that an activity unrelated to an endocrine disrupting effect influenced them.

VTG in male fish is a sensitive biomarker to identify that fish have been exposed to estrogenic (Esr agonistic) chemicals and thus has been frequently and widely used in laboratory and field studies for EDCs (Hara et al., 2016; Harries et al., 1997; Sumpter & Jobling, 1995; Wheeler, Gimeno, Crane, Lopez‐Juez, & Morritt, 2005; Yamanaka et al., 1998). In Japanese medaka, VTG induction level is generally assessed by either measuring the amount of VTG protein in blood (Chikae, Ikeda, Hasan, Morita, & Tamiya, 2004; Tabata et al., 2003) or liver extraction (Flynn et al., 2017; Nakamura et al., 2014; Seki et al., 2002), for example, by ELISA methods, or determining the amount of VTG mRNA expression in liver sample, for example, by quantitative polymerase chain reaction (qPCR) methods (Flynn et al., 2017; Lee, Jeon, Na, Choi, & Park, 2002). In the present study, hepatic VTG concentrations were quantitatively determined using two types of ELISA kits for which the intravariability and the intervariability had been assessed (Tatarazako et al., 2004).

In the Esr agonist studies, the VTG levels in male medaka exposed to E2, EE2, and PHT were significantly and concentration dependently elevated and far exceeded the levels in control females at the middle and/or the highest exposure concentrations. For these three chemicals, the LOECs on VTG induction in male were 22.1 ng/L (8.1 × 10^−11^ M), 17.8 ng/L (6.0 × 10^−11^ M), and 96.5 μg/L (5.9 × 10^−7^ M), respectively, and were more than 8,000 times (E2 and EE2) or 8 times (PHT) smaller than the LOEC for CMP (108 μg/L = 7.4 × 10^−6^ M). Regarding long‐term exposure effects of Esr agonists on Japanese medaka, Flynn et al. (2017) reported that the LOECs on male VTG induction and reproduction (fecundity or fertility) for E2 and CMP, obtained from the MMT studies, were both 28 ng/L and 345 and >345 μg/L, respectively. Likewise, as to the effects of PHT on Japanese medaka, Seki et al. (2003) reported that the LOECs on reproductive impairment and VTG induction in F0 generation (i.e., in the medaka continuously exposed to the test chemical after fertilization) were 224 and ≤51.1 μg/L, respectively in full life cycle test (FLCT). Furthermore, EE2 significantly elevated male VTG and reduced fertility in F0 generation even at 9.26 ng/L in FLCT (MOE, 2006). These results indicated that the sensitivity of male VTG is comparable between the FSTRA and the long‐term tests such as FLCT, MMT, and MEOGRT, although the endpoints related to reproductive status are rather less sensitive in the screening assay than in the definitive tests, especially with regard to Esr agonistic activities. The relationship of effective concentrations of the four Esr agonists were quite similar between the FSTRA and the agonist assay of RGA with mEsr1. As shown above, in the FSTRA, the LOECs (in molarity) for male VTG were almost the same for EE2 and E2, and the LOECs for PHT and CMP were 14,000 and 110,000 times higher than that of E2, respectively. In the results from the agonist assays, the EC_50_ was slightly smaller for EE2 than that of E2, as with the FSTRA, and the EC_50_s for PHT and CMP were 990 and 6,200 times higher than E2, respectively (Table 7). These results demonstrate that estrogenic potency in vivo is predictable from in vitro data in comparison of effective concentration rankings of Esr agonist chemicals for in vivo LOECs and in vitro EC_50_s (Lange et al., 2012).

A significant VTG induction was also found in the male medaka exposed to Esr antagonists. A selective Esr modulator, TAM, caused both the VTG induction in males and the VTG reduction in females significantly at the same concentration, 10.0 μg/L. Flynn et al. (2017) reported that the LOECs for TAM were 10 μg/L on reproduction (fertility) and 1.3 μg/L on female VTG (reduction) in MMT; hence, it is suggested that the sensitivity of reproduction to Esr antagonistic activity in FSTRA is not much different from long‐term exposure testing. As regards to the Esr agonistic activity, the FSTRA suggested that the LOEC on female VTG for TAM (10.0 μg/L) was 70 times lower than that for RAL (721 μg/L), whereas the antagonist assay of mEsr1 RGA indicated that the IC50 for TAM (0.14 μM) was more than 50 times higher than that of RAL (0.0026 μM). Although the results of only two chemicals, these might be suggested that uncertainties remain in extrapolating from in vitro to in vivo toxicity, due to differences in, for example, the physicochemical properties or the external and internal concentrations of the chemicals tested (Groothuis et al., 2015; Stadnicka‐Michalak, Tanneberger, Schirmer, & Ashauer, 2014). VTG reduction in female fish is also a sensitive indicator for detecting effects by chemicals with aromatase/steroidogenesis inhibitory activity (Panter et al., 2004; Villeneuve et al., 2009).

The formation of PP on anal fin is one of the masculine SSCs in Japanese medaka (Iguchi, Ogino, Miyagawa, Yatsu, & Tatarazako, 2019; Oka, 1931) and is an easily quantifiable in vivo assay endpoint. The molecular mechanisms in their development have been elucidated (Ogino et al., 2014). In the AR agonist studies, MT, DHT, and TRB induced PP on female anal fins, and the numbers were concentration dependently increased. The LOECs on PP formation in females for these three AR agonists were 20.1 ng/L, 1.03 μg/L, and 26.8 ng/L, respectively, and were the most sensitive in the endpoints for FSTRA. In the long‐term testing (e.g., FLCT and MMT), induction of SSC in females and reduction of reproduction were caused at 9.98 ng/L of MT (Seki et al., 2004), and at 32 and 13 ng/L, respectively for TRB (Flynn et al., 2017). These results indicated that SSC induction in female Japanese medaka has the same sensitivity between short‐term and long‐term exposure, and thus, there is a possibility that the LOEC from the short‐term screening assay is indicative of the suspected effects on reproduction in long‐term exposure, as well as the VTG in male for Esr agonists.

A reduction of SSC in males is an endpoint to identify the antiandrogenic (AR antagonistic) effect of test chemicals in a short‐term assay. Panter et al. (2004) reported that FLT at 938.6 μg/L significantly reduced the number of nuptial tubercles in male fathead minnow in a 21‐day exposure and the validation study of FSTRA using fathead minnow demonstrated that all the three participating laboratories detected a statistically significant decrease in nuptial tubercle number in the male fish exposed to VCZ at the nominal concentration of 900 μg/L (US EPA, 2007). On the other hand, a significant effect on male SSC was detected for neither FLT nor VCZ in the present study. As for the effects of AR antagonist on Japanese medaka, Flynn et al. (2017) reported that the LOECs on reproduction (fecundity and fertility) and SSC in females were 253 and 33 μg/L, respectively for VCZ in the MMT study. Furthermore, both FLT and VCZ inhibited the mAR*β*‐mediated transactivational activity induced by 50‐nM 11KT in the antagonist assay of RGA using mAR*β* (Table 7). In mature male medaka, a 21‐day exposure period might not be enough to invisibly degenerate the PPs that had already formed as a branching bone nodule from bone segments in anal fin rays (Ogino et al., 2014). Hence, to adequately detect AR antagonistic activity in vivo screening, it is necessary to establish a more sensitive novel test method, for example, using juvenile medaka (Nakamura et al., 2014).

In conclusion, the present study demonstrated that the FSTRA (TG229) using Japanese medaka is applicable in identifying the effects of chemicals with Esr agonistic, Esr antagonistic, and AR agonistic potency on the reproduction of fish, as well as steroidogenesis inhibitory activity, though the assay suggested an insensitivity to AR antagonists. In addition, the Japanese medaka demonstrated the effects of the progestin, LNG, which masculinized and reduced reproductive activity in females by activating ARs in the FSTRA, similar to the reports on other small test fish (Ellestad et al., 2014; Frankel et al., 2016; Svensson et al., 2013). Regarding the endpoints, VTG and SSC were sensitive to the activities of Esr agonists and antagonists, and AR agonists, respectively, and their LOECs obtained from the FSTRA might be helpful in inferring the effect concentration on reproduction in long‐term exposure. Furthermore, the spawning status, such as the change in daily fecundity over the exposure duration, will support identifying the MOA for the test chemical, though it is necessary to take into account the possibility of toxic effects not interacting with the endocrine system, such as a surfactant action. On the other hand, a fairly high variability among the controls for the 16 chemical studies was found in female VTG levels and fecundity and suggested that comparison between the treatment and the control fish should be limited within the same study for these endpoints. In order to enrich the guidance on interpretation of the screening assay, including the in vitro RGA, it will be necessary to constantly extend and update its knowledge base, for example, incorporating data from the FSTRA (TG229) conducted under the EXTEND 2016 Program.

### DATA AVAILABILITY STATEMENT

Data openly available in a public repository that issues datasets with DOIs.
